# Resistance Mutations in CLL: Genetic Mechanisms Shaping the Future of Targeted Therapy

**DOI:** 10.3390/genes16091064

**Published:** 2025-09-10

**Authors:** Samantha Sekeres, Erica N. Lamkin, Eduardo Bravo, Allison Cool, Justin Taylor

**Affiliations:** 1 Sylvester Comprehensive Cancer Center, University of Miami Miller School of Medicine, Miami, FL 33136, USA; sds247@miami.edu (S.S.); enl55@med.miami.edu (E.N.L.); exb1333@med.miami.edu (E.B.J.); axc3213@miami.edu (A.C.); 2 College of Engineering, University of Miami, Miami, FL 33146, USA; 3 Medical Scientist Training Program, University of Miami Miller School of Medicine, Miami, FL 33136, USA; 4 Sheila and David Fuente Graduate Program in Cancer Biology, University of Miami Miller School of Medicine, Miami, FL 33136, USA

**Keywords:** chronic lymphocytic leukemia (CLL), targeted therapy, BTK inhibitors, BCL2 inhibitors, resistance mechanisms, precision medicine, PROTAC degraders, measurable residual disease (MRD)

## Abstract

Chronic lymphocytic leukemia (CLL) is the most common type of leukemia in Western populations and remains incurable despite significant therapeutic advancements. Over the past decade, the treatment landscape has evolved from traditional chemoimmunotherapy to targeted oral agents, including Bruton’s tyrosine kinase inhibitors (BTKis) and BCL2 inhibitors (BCL2is), which have demonstrated superior efficacy and tolerability, especially in elderly patients. Venetoclax, a BCL2i, induces apoptosis in CLL cells through selective inhibition of the anti-apoptotic BCL2 protein, while BTKis, such as ibrutinib and its next-generation analogs, disrupt B-cell receptor signaling critical to CLL cell survival. However, resistance to both drug classes has emerged, including mutations in BTK and BCL2, prompting the exploration of novel therapeutic strategies. This review outlines the molecular basis and clinical implications of these resistance mechanisms, as well as emerging therapeutic solutions, including non-covalent BTKis like pirtobrutinib and BTK-targeting PROTAC degraders such as BGB-16673 and NX-2127. Additionally, we discuss promising combination therapies incorporating BTKis, BCL2is, and anti-CD20 monoclonal antibodies. Finally, we highlight the growing role of measurable residual disease (MRD) as a biomarker to guide treatment duration and evaluate therapeutic success. As resistance mechanisms continue to emerge, tailoring therapy based on underlying biology will be critical to sustaining disease control and enhancing outcomes in patients with CLL.

## 1. Introduction

Chronic lymphocytic leukemia (CLL), occurring most often in elderly adults, is the most prevalent type of leukemia in Western populations [1]. CLL is characterized by the clonal expansion of B-cells, confirmed by the presences of B-cell markers, aberrant CD5 expression, and monoclonality [1,2]. The severity of CLL varies from case to case and, therefore, so does the treatment plan. While currently available treatments often lead to disease remission, CLL remains an incurable disease [1].

A diagnosis of CLL is verified by sustained lymphocytosis (>0.5 × 10^9^ lymphocytes/L), predominance of small, mature lymphocytes in the blood smear, and classification of cells via immunophenotyping [3]. Because of the nature of CLL, it can be identified before it is symptomatic. Around 70% of patients with CLL are diagnosed when routine examinations reveal unexplained lymphocytosis although no other symptoms are present [4,5].

Following diagnosis, median survival of patients with CLL can range from several months to over 10 years, without treatment [6]. The Binet and Rai staging systems are used to measure progression of disease, basing the relative risk on physical examination of lymph nodes and blood cell counts [7,8].

The treatment landscape for CLL has changed drastically since 2014, when the frontline therapy was chemoimmunotherapy (CIT). Fludarabine, cyclophosphamide, and rituximab (FCR), bendamustine and rituximab (BR), and chlorambucil were the most common treatment regimens, with FCR given to younger patients (under 65 years), BR given to older patients (between 65 and 75 years), and single-agent chlorambucil given to elderly patients (over 75 years) or those with comorbidities. In the last 10 years, these aggressive treatments have been largely replaced by targeted oral inhibitors due to increased tolerability and efficacy [9].

## 2. Emergence of Targeted Therapies

Targeted therapies have revolutionized the treatment of CLL. Both Bruton’s tyrosine kinase inhibitors (BTKis) and B-cell lymphoma 2 inhibitors (BCL2i) have shown great success as targeted oral inhibitors, rewriting the standard of care in CLL [10]. Given the older demographic of the majority of CLL patients, targeted therapies have provided a significantly less toxic treatment option, compared to the previous standard of care, CIT, that also has shown more success in allowing patients to reach remission [11].

### 2.1. B-Cell Lymphoma 2 Inhibitors

**Figure 1:**
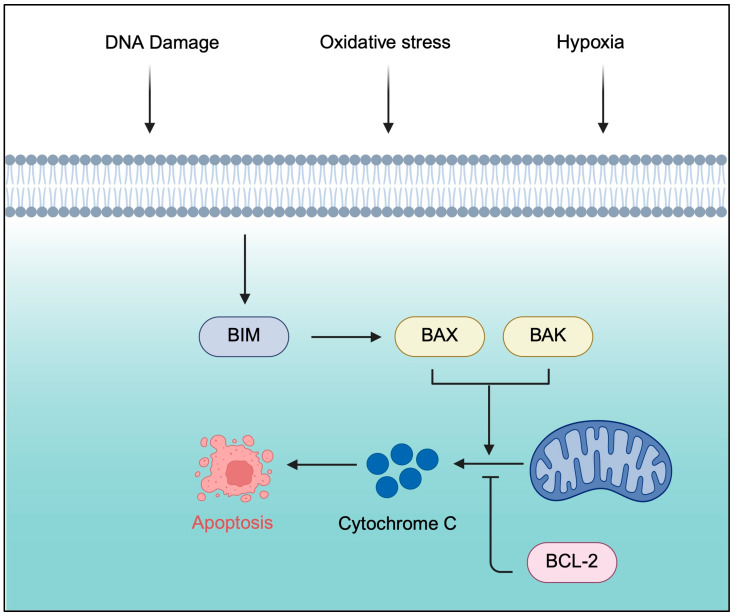
BCL2 is a regulator of apoptosis in B cells. This figure depicts the role of BCL2 interacting mediator (BIM), BAX, BAK, and BCL2 in response to stimuli that induce apoptosis. BCL2i target this pathway to induce apoptosis in cells that depend on BCL2 for survival. Image created using BioRender.com [12].

BCL2i were developed due to the role that BCL2 proteins play in cell death regulation. BCL2 proteins are a protein family that include many survival-determining proteins, with both pro- and anti-apoptotic functions [12]. The BCL2 homology 3 (BH3) proteins are a subgroup that induces apoptosis, as shown in Figure 1; among these are BCL2-associated X proteins (BAXs) and BCL2 antagonists/killers (BAKs). However, BCL2 itself is part of the pro-survival subgroup [13]. Overexpression of anti-apoptotic proteins like BCL2 promotes the survival of malignant B cells and contributes to the pathogenesis of B-cell malignancies, including CLL [14]. The first BCL2i, venetoclax, was FDA-approved in 2016, following success in a phase 2 clinical trial (NCT01889186) [15]. Venetoclax, a reversible inhibitor, delivers its therapeutic effect by mimicking the BH3 domain and engaging the P2 and P4 sites of BCL2 for effective inhibition, mainly through hydrogen bonding and hydrophobic interactions. This displaces BH3-only proteins which allows apoptosis to occur [16,17]. Venetoclax binds to BCL2 and selectively isolates it from BIM, allowing subsequent activation of BAX/BAK, causing the apoptotic pathway to activate [18,19].

Venetoclax alone did not demonstrate high rates of complete remission (CR), which warranted the examination of combination therapies [20]. Venetoclax was therefore studied in combination with anti-CD20 monoclonal antibodies: rituximab and obinutuzumab. Several clinical trials demonstrated the efficacy of this combination, with overall response rates ranging from 84.7% (CLL14 [21]) to 100% (NCT01685892 [22]) and undetectable MRD (uMRD) from 62.4% (MURANO [23]) to 92.9% (HOVON139/GIVE NTR604 [24]).

Following treatment with venetoclax, acquired mechanisms of resistance can develop through increased expression of other anti-apoptotic proteins. Among these are BCL-xL and BCL-2A1 induced resistance. Additional mechanisms of resistance, induced through increased activation of NF-kB signaling, have also been implicated in resistance to venetoclax in CLL: interleukin 10, CD40L, and stimulation of TLR9 by unmethylated DNA [25,26]. Other identified mutations involve changes in amino acids: aspartic acid at position 103 with tyrosine (D103Y) and glycine at position 101 with valine (G101V). The D103Y mutation occurs within the BH3 binding pocket of BCL2, the replacement of aspartic acid with tyrosine inhibits venetoclax from binding as the bulkier tyrosine extends into the binding pocket. The G101V mutation causes a conformational change in the BH3 binding pocket, hindering venetoclax from binding by overcrowding residues E152, V148, F104, and Y18 [27].

The first course of action to overcome this resistance is the use of combination therapies. In acute myeloid leukemia (AML), previously resistant cells became venetoclax-sensitive again following treatment with myeloid cell leukemia-1 inhibitor, identified in the clinical trial, VU661013 [28,29,30]. The success of combination therapies in other hematologic malignancies strongly encourages the exploration of similar strategies in CLL patients who develop resistance to targeted treatments.

### 2.2. Bruton’s Tyrosine Kinase Inhibitors

Bruton’s tyrosine kinase (BTK) became a key target in CLL due to its role in B-cell proliferation and its proven need for selectivity. BTKis can be broadly categorized into covalent BTKis (cBTKis) and non-covalent BTKis (ncBTKis), either through irreversible covalent binding or reversible non-covalent binding. Ibrutinib, a cBTKi, was the first widely approved small-molecule BTK inhibitor for the treatment of relapsed/refractory (R/R) CLL [31]. Ibrutinib was FDA-approved in 2016 following the phase 3 RESONATE study, which found that treatment with ibrutinib was associated with significantly longer progression-free survival (PFS) and an increased overall survival (OS) rate as compared to chlorambucil [32,33]. Ibrutinib inhibits BTK by binding covalently to the cysteine 481 (C481) residue of BTK to block phosphorylation and downstream B-cell receptor signaling, critical for the survival of many B-cell malignancies like CLL [34]. However, its limited selectivity results in off-target effects on other kinases with cysteines in the same alignment (e.g., EGFR, ITK, JAK3, ErbB2, and TEC), leading to side effects in patients [35,36]. The need for more specific BTKis emerged from patients with X-linked agammaglobulinemia (XLA). This immunodeficiency results in an absence of BTK activity; however, both patients with and without XLA saw heightened risk of bleeding following treatment with ibrutinib. It can be deduced that the risk was not a result of inhibition of BTK but rather the erratic activity caused by ibrutinib [36]. Second-generation cBTKis, such as acalabrutinib, tirabrutinib and zanabrutinib, were developed with improved selectivity towards BTK to mitigate off-target effects [37,38]. This medicinal chemistry strategy was effective until point mutations arose, preventing covalent binding and rendering these inhibitors ineffective in affected patients [32].

Resistance to cBTKi emerged through mutations at the drug binding site, most notably the C481S mutation, which disrupts the covalent interaction and reduces drug efficacy. However, it was found that a new class of BTKi, non-covalent BTK inhibitors, could be employed to overcome resistance caused by mutations such as C481S by binding reversibly to BTK at alternate sites, maintaining therapeutic potential, even when covalent inhibitors are no longer effective [39]. ncBTKis harness the chemical space within the active site to engage in drug–target interactions to effectively inhibit BTKs. Specifically, ncBTKis bind to BTKs using intramolecular interactions (hydrogen bonds, hydrophobic interactions, and ionic bonds) as opposed to cBTKis that bind directly to the BTK-C481 residue [39,40]. Thus, ncBTKis were designed to overcome the BTK-C481S resistance mutation for effective therapeutic effect [41,42]. This strength is seen in pirtobrutinib, the most successful ncBTKi that has been developed and explored thus far [43].

**Figure 2:**
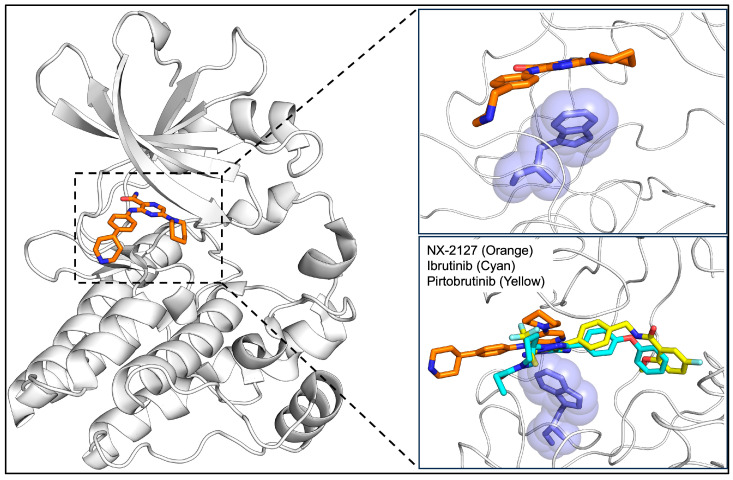
Structures of inhibitors bound to BTK (PDB: 8GC8 [40], 5P9J [46], 8FLL [52]). The top panel depicts the binding mode of NX-2127 to the L528W mutant. The bottom panel compares the binding modes of NX-2127 (orange), ibrutinib (cyan), and pirtobrutinib (yellow). The L528W mutation (shown in slate) introduces a bulky steric hindrance that prevents the binding of ibrutinib and pirtobrutinib, rendering them ineffective. In contrast, NX-2127 accommodates the steric change, maintaining its ability to bind, inhibit, and promote degradation of BTK [53,54,55,56].

Several mutations in BTK have been identified that confer resistance to covalent and non-covalent BTKis: C481S, L528W, and T474I [44,45]. After exposure to cBTKis, subsequent mutations can develop that additionally show resistance to ncBTKis; these mutations have been found to aggregate in the kinase domain of BTK, conferring resistance to both cBTKis and ncBTKis [44,46]. For example, in patients who already carry a C481S mutation and have subsequently switched therapies from cBTKi to ncBTKi, the L528W mutation has been observed to emerge, conferring resistance to pirtobrutinib. This suggests that L528W arises as a distinct resistance mechanism to ncBTKi rather than as a consequence of the C481S mutation itself [47,48]. L528W is categorized as a kinase-impaired mutation, as it blocks the catalytic activity of BTK while still allowing downstream BCR signaling and activation of the AKT pathway [44,48]. A bioinformatics analysis found that the L528W mutation may reduce conformational stability and increase the flexibility of BTK, leading to structural destabilization. The study suggested that the sidechain of Trp528 stuck out towards the active center of BTK, causing steric hindrance, pushing ibrutinib away from the C481 region, and inhibiting the formation of a covalent bond [49]. Additionally, an increase in free energy caused by the L528W mutation was identified; this is thought to be a leading factor resulting in the decreased binding affinity of BTKis [40,50,51]. The crystal structure of L528W was confirmed in 2024 and is depicted in Figure 2 [40].

## 3. Evolving Therapies

### 3.1. Combination Therapies

Due to the initial efficacy of BTKi and BCL2i as independent therapeutic regimens, and the resulting resistance, the two targeted therapies began being investigated in combination [57,58]. A correlative study that analyzed CLL cells obtained ex vivo and in vitro found that when ibrutinib and venetoclax were given together there was an observed reduction in the mitochondrial capacity to produce energy, disrupting cellular signaling pathways [59]. This combination also allowed for time-limited treatment instead of continuous treatment as typically required in BTKi only regimens, as seen in patients in the CAPTIVATE trial [60].

Previously, anti-CD20 therapies have been combined with chemotherapies as a doublet therapeutic regimen. In the phase 3 GLOW trial, chlorambucil-obinutuzmab was compared with ibrutinib-venetoclax combination therapies to conclude that the BTKi-BCL2i combination was superior for PFS in elderly and comorbid patients [21]. The superiority of the ibrutinib–venetoclax combination was defined through improved potency and duration of response [21,23].

The CAPTIVATE study, in which BTKi and venetoclax were given in combination found that even with patients who were not treatment naïve, the combination of the two therapies provided valuable results [58]. Additionally, the doublet therapy provided encouraging results for patient populations with high genomic risk; patients with unmuted immunoglobulin heavy chains (IGHs) and TP53 mutations. Combining the two therapies allowed some patients to overcome molecular resistance to BTKis, encouraging the success of the combination [57,58,61,62].

Furthermore, novel therapy regimens have looked at the therapeutic potential of combining BTKis, BCL2i, and the anti-CD20 monoclonal antibody, obinutuzumab. Several triplet therapies are being tested in clinical trials; among these are the ibrutinib, venetoclax, and obinutuzumab (IVO) combined therapy, currently in various phase 1–3 studies [63,64]. The phase 3 trial, A041702, compares IVO to ibrutinib and obinutuzumab doublet therapy, finding that IVO showed an uMRD of 86.6% and a CR of 68.5% after 14 cycles [65]. Acalabrutinib, venetoclax, and obinutuzumab (AVO) combination has also been investigated in a phase 2 trial, finding that in cases of CR of uMRD, AVO could be stopped after 15 cycles, or in cases of any uMRD response, after 24 cycles; in all other cases, acalabrutinib use continued after the administration of the triplet regimen [66].

One study combined an ncBTKi, pirtobrutinib, with venetoclax and obinutuzumab (PVO), finding a high proportion of bone marrow (BM) uMRD after 6 and 12 months of the triplet therapy. After 6 months, BM and peripheral blood (PB) showed uMRD6 (1 in 10^6^ leukocytes) in 65% and 79% of patients, respectively. After 12 months, BM and PB showed uMRD6 in 81% and 89% of patients, respectively [67].

A significant drawback of the rigorous triplet therapy is the proportion of patients that have resulting neutropenia and thrombocytopenia [63,67]. Grade 3+ neutropenia occurred in 48% and 43% of IVO and AVO trial regimens, respectively. Grade 3+ thrombocytopenia was also found in 16% and 27% of IVO and AVO patients, respectively [63]. In the PVO study, grade 3+ neutropenia and thrombocytopenia were seen in 58% and 18% of patients, respectively [67].

### 3.2. BTK PROTAC Degraders

Given the persistent clinical challenge of acquired BTK mutations in response to the aforementioned targeted therapies, BTK proteolysis-targeting chimera (PROTAC) degraders have emerged as a promising therapeutic avenue [68]. This novel class of drug possesses alternative mechanisms of action and improved kinome selectivity, shown in Figure 2, that does not require continual structural modification [53]. PROTACs represent a new paradigm in drug discovery by enabling the complete removal of the protein of interest (POI) from the cell via proteasomal degradation. PROTACs are heterobifunctional molecules consisting of three important parts. First, a ligand binding to the protein of interest. This targeting ligand is often an inhibitor, and its main role is to bind to the target of interest. The ligand is connected via a linker to a second ligand which recruits an E3 ligase, such as cereblon or von Hippel–Lindau (VHL). The heterobifunctional PROTAC results in the formation of a ternary complex which leads to polyubiquitination of the desired target by transferring ubiquitin molecules from the ubiquitin-conjugating enzyme known as E2 to the lysine residue on the target protein. The polyubiquitination of the target protein is then recognized by the proteosome, leading to its degradation [53,54,55,56]. BTK degraders are currently being studied in clinical trials, of which BGB-16673, NX-2127, and NX-5948 have proven safety and efficacy in phase 1 [40,69].

**Figure 3:**
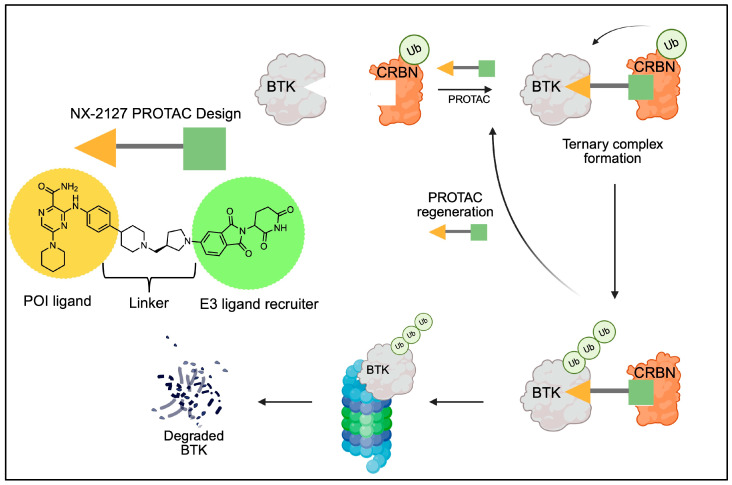
Degradation mechanism of NX-2127, a BTK PROTAC. PROTACs are heterobifunctional molecules formed by ligand binding to the protein of interest (yellow) connected by a linker (gray) to an E3 ligase recruiter (green). Upon ubiquitination, BTK is targeted for proteosomal degradation, and the PROTAC is released and engages in subsequent degradation cycles. Image created using BioRender.com [53,54,55,56].

BGB-16673 functions using the BTK-binding and E3 ubiquitin ligase-binding domains degrading both wild-type (WT) BTK as well as mutated BTK. Alternatively, NX-2127 and NX-5948 degrade BTK through the recruitment of the cereblon (CRBN) E3 ubiquitin ligase complex [69,70]. A recent study found that treatment of CLL cells with both NX-2127 and NX-5948 degraded BTK without inhibiting the growth or activity of CD3^+^ T cells [70]. Additionally, NX-2127 degrades CRBN neosubstrates Aiolos (IKZF3) and Ikaros (IKZF1), increasing T-cell activation and fostering anti-tumor effects [40,71]. NX-2127, the mechanism of which is depicted in Figure 3, also results in decreased levels of the mutant C481S-BTK [40,72].

The ability of BTK degraders to completely break down BTK, inhibiting its function and the proliferation of the BCR complex, shows promise in CLL patients, specifically those with mutations that confer resistance to BTKis [72].

However, a recent case study identified a mutation that could cause resistance to BGB-16673:BTK-A428D [73]. This study followed a male who was diagnosed at 66 years old and developed resistance to a multitude of BTKis, and was ultimately treated with BGB-16673. The patient had CLL with unmutated IGHV and subsequently underwent a series of therapeutic regimens, including BR, VO, IVO, and acalabrutinib, achieving varying levels of remission and adverse events throughout the treatment course. Next-generation sequencing (NGS) revealed a BTK C481S mutation as well as a TP53 mutation. Following this analysis, the patient underwent treatment with pirtobrutinib. Later NGS of his bone marrow aspirate revealed another BTK mutation, T474I, that led to the eventual initiation of BTK degrader therapy, BGB-16673, in a clinical trial. He came off the study after four months due to the progression of his disease, with NGS showing another BTK mutation, BTK-A428D [73]. Similarly to L528W, the BTK-A428D mutation is hypothesized to be kinase-impaired [74]. Wong et al. hypothesized that the BTK degrader applied selective pressure that influenced the development of the A428D mutation [73]. Notably, no functional studies were performed to show that A428D confers resistance to BTK degraders, and it is still ambiguous whether this mutation is indeed a cause of resistance (Table 1).

## 4. Conclusions and Future Directions

The treatment landscape for CLL is progressing rapidly, presenting promise for widely available, effective treatments, with strategies to monitor disease progression. The focus on BTK-targeted therapies does not appear as though it will fade as resistance increases, but rather the same target will be approached through different mechanisms in response to each mutation that emerges. However, there is no existing curative therapy for CLL, and therefore, treatment duration remains indefinite, barring adverse events [78]. The uncertainty with longevity of treatment promotes the use of combination therapies, with established cycles, as a frontline therapy [79]. As the treatment landscape continues to evolve, it is integral to continue characterizing resistance mechanisms to guide the future of CLL therapies.

As there is uncertainty with when therapies can be discontinued, physicians and researchers turn to methods through which they can track disease progression and efficacy. Detection of MRD is an integral tool for measuring disease progression. MRD quantifies the number of CLL cells detected within a sample, with uMRD being the target for effective therapies [80,81]. As previously discussed, venetoclax and ibrutinib were combined as a therapeutic regimen in the CAPTIVATE (PCYC-1142) study [60,82]. In this study, patients underwent fixed-duration treatment regimens with MRD assessed at established timepoints. The phase 2 trial found that FD patients saw increased uMRD after 12 cycles of the therapy and confirmed that uMRD encouraged the potential for FD treatment [58].

Key focuses of MRD-guided approaches to therapy include the method of detection, detection endpoint, and comparison with PFS or OS [80]. Crucial and ongoing clinical trials have used uMRD as a guide for success as well as discontinuation of treatment. Flow cytometry (FC), immunoglobulin heavy-chain polymerase chain reaction (IgH-PCR), and next-generation sequencing (NGS) are all effective methods of measuring uMRD [83,84,85,86]. IgH-PCR can be paired with allele-specific oligonucleotide (ASO) primers, both of which are considered the most precise techniques for MRD detection, with a sensitivity of up to MRD6 [81]. FC is the most commonly used technique, however, due to its relative accessibility [84]. NGS was implemented in the phase 3 GLOW trial, finding that the targeted therapy combination was superior to the CIT combination []. NGS is currently being implemented with the clonoSEQ platform in CLL [87].

MRD can be implemented as a tool for making decisions about treatment regimens and disease progression, provided it is used at the right time and in the right way to enhance patient outcomes [88].

## 5. Methods

This narrative review was conducted through a comprehensive literature search using Google Scholar and PubMed. Search terms included ‘CLL’, ‘BCL2 inhibitors’, ‘BTK inhibitors’, ‘CLL therapeutic resistance mutations’, ‘BTK degraders’, ‘combination therapeutics in CLL’, and ‘measurable residual disease in CLL’. Articles were scanned manually and selected for relevance and currentness, applying a date range from 1995 to 2025 [57].

## Figures and Tables

**Table 1:** Resistance mechanisms to BTK-targeting therapies.

Mutation	Therapeutic Resistance	Mechanism of Resistance	Therapy that Overcomes Resistance	References
C481S	Ibrutinib/Zanubrutinib/Acalabrutinib	Gatekeeper; disrupts irreversible binding of cBTKi through alteration of 481 residue	Pirtobrutinib/BTK Degraders	[46,75]
L528W	Zanubrutinib/Pirtobrutinib	Kinase-impaired; blocks activity of BTK while continuing downstream signaling of the BCR pathway	BTK Degraders	[44,76]
T474I	Acalabrutinib/Pirtobrutinib	Gatekeeper; alters 474 residues to prevent binding	BTK Degraders	[43,44,46,76,77]
A428D	Ibrutinib/Zanubrutinib	A428D mutation has been associated with resistance, but the mechanism has not been determined or characterized		[76,77]
	Pirtobrutinib	Kinase-impaired; blocks activity of BTK while continuing downstream signaling of the BCR pathway		[44]

## Data Availability

No new data were created or analyzed in this study. Data sharing is not applicable to this article.

## Abbreviations

The following abbreviations are used in this manuscript:

CLL	Chronic lymphocytic leukemia
CIT	Chemoimmunotherapy
BR	Bendamustine and rituximab
BTKi	Bruton’s tyrosine kinase inhibitor
BCL2i	B-cell lymphoma 2 inhibitor
MRD	Measurable residual disease
BH3	B-cell lymphoma 2 homology 3
BAX	B-cell lymphoma 2-associated X proteins
BAK	B-cell lymphoma 2 antagonist/killer
BIM	B-cell lymphoma 2-interacting mediator
uMRD	Undetectable measurable residual disease
AML	Acute myeloid leukemia
BTK	Bruton’s tyrosine kinase
cBTKi	Covalent Bruton’s tyrosine kinase inhibitor
ncBTKi	Non-covalent Bruton’s tyrosine kinase inhibitor
R/R	Relapsed/refractory
PFS	Progression-free survival
OS	Overall survival
XLA	X-linked agammaglobulinemia
IGH	Immunoglobulin heavy chain
IVO	Ibrutinib, venetoclax, and abinutuzumab
AVO	Acalabrutinib, venetoclax, and obinutuzumab
PVO	Pirtobrutinib, venetoclax, and obinutuzumab
BM	Bone marrow
PB	Peripheral blood
PROTAC	Proteolysis-targeting chimera
POI	Protein of interest
VHL	Von Hippel–Lindau
CRBN	Cereblon
FC	Flow cytometry
IgH-PCR	Immunoglobulin heavy-chain polymerase chain reaction
NGS	Next-generation sequencing
ASO	Allele-specific oligonucleotide

## References

[1] Dighiero G. (2005). CLL Biology and Prognosis. Hematology.

[2] Rai K.R., Jain P. (2016). Chronic lymphocytic leukemia (CLL)—Then and now. Am. J. Hematol..

[3] Alaggio R., Amador C., Anagnostopoulos I., Attygalle A.D., Araujo I.B.d.O., Berti E., Bhagat G., Borges A.M., Boyer D., Calaminici M. (2022). The 5th edition of the World Health Organization Classification of Haematolymphoid Tumours: Lymphoid Neoplasms. Leukemia.

[4] Shadman M. (2023). Diagnosis and Treatment of Chronic Lymphocytic Leukemia: A Review. JAMA.

[5] Abrisqueta P., Pereira A., Rozman C., Aymerich M., Giné E., Moreno C., Muntañola A., Rozman M., Villamor N., Hodgson K. (2009). Improving survival in patients with chronic lymphocytic leukemia (1980–2008): The Hospital Clínic of Barcelona experience. Blood.

[6] Mauro F.R., Foa R., Giannarelli D., Cordone I., Crescenzi S., Pescarmona E., Sala R., Cerretti R., Mandelli F. (1999). Clinical Characteristics and Outcome of Young Chronic Lymphocytic Leukemia Patients: A Single Institution Study of 204 Cases. Blood.

[7] Rai K.R., Sawitsky A., Cronkite E.P., Chanana A.D., Levy R.N., Pasternack B.S. (1975). Clinical Staging of Chronic Lymphocytic Leukemia. Blood.

[8] Binet J.L., Auquier A., Dighiero G., Chastang C., Piguet H., Goasguen J., Vaugier G., Potron G., Colona P., Oberling F. (1981). A new prognostic classification of chronic lymphocytic leukemia derived from a multivariate survival analysis. Cancer.

[9] Kay N.E., Hampel P.J., Van Dyke D.L., Parikh S.A. (2022). CLL update 2022: A continuing evolution in care. Blood Rev..

[10] Karr M., Roeker L. (2023). A History of Targeted Therapy Development and Progress in Novel–Novel Combinations for Chronic Lymphocytic Leukemia (CLL). Cancers.

[11] Smolej L., Vodárek P., Écsiová D., Šimkovič M. (2021). Chemoimmunotherapy in the First-Line Treatment of Chronic Lymphocytic Leukaemia: Dead Yet, or Alive and Kicking?. Cancers.

[12] Kluck R.M., Bossy-Wetzel E., Green D.R., Newmeyer D.D. (1997). The Release of Cytochrome c from Mitochondria: A Primary Site for Bcl-2 Regulation of Apoptosis. Science.

[13] Kvansakul M., Yang H., Fairlie W.D., Czabotar P.E., Fischer S.F., Perugini M.A., Huang D.C.S., Colman P.M. (2008). Vaccinia virus anti-apoptotic F1L is a novel Bcl-2-like domain-swapped dimer that binds a highly selective subset of BH3-containing death ligands. Cell Death Differ..

[14] Cimmino A., Calin G.A., Fabbri M., Iorio M.V., Ferracin M., Shimizu M., Wojcik S.E., Aqeilan R.I., Zupo S., Dono M. (2005). miR-15 and miR-16 induce apoptosis by targeting BCL2. Proc. Natl. Acad. Sci. USA.

[15] Stilgenbauer S., Eichhorst B., Schetelig J., Coutre S., Seymour J.F., Munir T., Puvvada S.D., Wendtner C.-M., Roberts A.W., Jurczak W. (2016). Venetoclax in relapsed or refractory chronic lymphocytic leukaemia with 17p deletion: A multicentre, open-label, phase 2 study. Lancet Oncol..

[16] Bose P., Gandhi V., Konopleva M. (2017). Pathways and mechanisms of venetoclax resistance. Leuk. Lymphoma.

[17] Birkinshaw R.W., Gong J.-N., Luo C.S., Lio D., White C.A., Anderson M.A., Blombery P., Lessene G., Majewski I.J., Thijssen R. (2019). Structures of BCL-2 in complex with venetoclax reveal the molecular basis of resistance mutations. Nat. Commun..

[18] Anderson M.A., Deng J., Seymour J.F., Tam C., Kim S.Y., Fein J., Yu L., Brown J.R., Westerman D., Si E.G. (2016). The BCL2 selective inhibitor venetoclax induces rapid onset apoptosis of CLL cells in patients via a TP53-independent mechanism. Blood.

[19] Souers A.J., Leverson J.D., Boghaert E.R., Ackler S.L., Catron N.D., Chen J., Dayton B.D., Ding H., Enschede S.H., Fairbrother W.J. (2013). ABT-199, a potent and selective BCL-2 inhibitor, achieves antitumor activity while sparing platelets. Nat. Med..

[20] Seymour J.F., Ma S., Brander D.M., Choi M.Y., Barrientos J., Davids M.S., Anderson M.A., Beaven A.W., Rosen S.T., Tam C.S. (2017). Venetoclax plus rituximab in relapsed or refractory chronic lymphocytic leukaemia: A phase 1b study. Lancet Oncol..

[21] Fischer K., Al-Sawaf O., Bahlo J., Fink A.-M., Tandon M., Dixon M., Robrecht S., Warburton S., Humphrey K., Samoylova O. (2019). Venetoclax and Obinutuzumab in Patients with CLL and Coexisting Conditions. N. Engl. J. Med..

[22] Flinn I.W., Gribben J.G., Dyer M.J.S., Wierda W., Maris M.B., Furman R.R., Hillmen P., Rogers K.A., Iyer S.P., Quillet-Mary A. (2019). Phase 1b study of venetoclax-obinutuzumab in previously untreated and relapsed/refractory chronic lymphocytic leukemia. Blood.

[23] Kater A.P., Owen C., Moreno C., Follows G., Munir T., Levin M.-D., Benjamini O., Janssens A., Osterborg A., Robak T. (2022). Fixed-Duration Ibrutinib-Venetoclax in Patients with Chronic Lymphocytic Leukemia and Comorbidities. NEJM Evid..

[24] Kersting S., Dubois J., Nasserinejad K., A Dobber J., Mellink C., van der Kevie-Kersemaekers A.-M.F., Evers L.M., de Boer F., Koene H.R., Schreurs J. (2022). Venetoclax consolidation after fixed-duration venetoclax plus obinutuzumab for previously untreated chronic lymphocytic leukaemia (HOVON 139/GiVe): Primary endpoint analysis of a multicentre, open-label, randomised, parallel-group, phase 2 trial. Lancet Haematol..

[25] Ryan C.E., Davids M.S. (2019). BCL-2 Inhibitors, Present and Future. Cancer J..

[26] Lee H.H., Dadgostar H., Cheng Q., Shu J., Cheng G. (1999). NF-kappaB-mediated up-regulation of Bcl-x and Bfl-1/A1 is required for CD40 survival signaling in B lymphocytes. Proc. Natl. Acad. Sci. USA.

[27] Tausch E., Close W., Dolnik A., Bloehdorn J., Chyla B., Bullinger L., Döhner H., Mertens D., Stilgenbauer S. (2019). Venetoclax resistance and acquired BCL2 mutations in chronic lymphocytic leukemia. Haematologica.

[28] Ramsey H.E., Fischer M.A., Lee T., Gorska A.E., Arrate M.P., Fuller L., Boyd K.L., Strickland S.A., Sensintaffar J., Hogdal L.J. (2018). A Novel MCL1 Inhibitor Combined with Venetoclax Rescues Venetoclax-Resistant Acute Myelogenous Leukemia. Cancer Discov..

[29] Leverson J.D., Zhang H., Chen J., Tahir S.K., Phillips D.C., Xue J., Nimmer P., Jin S., Smith M., Xiao Y. (2015). Potent and selective small-molecule MCL-1 inhibitors demonstrate on-target cancer cell killing activity as single agents and in combination with ABT-263 (navitoclax). Cell Death Dis..

[30] Lee T., Bian Z., Zhao B., Hogdal L.J., Sensintaffar J.L., Goodwin C.M., Belmar J., Shaw S., Tarr J.C., Veerasamy N. (2017). Discovery and biological characterization of potent myeloid cell leukemia-1 inhibitors. FEBS Lett..

[31] Byrd J.C., Furman R.R., Coutre S.E., Flinn I.W., Burger J.A., Blum K.A., Grant B., Sharman J.P., Coleman M., Wierda W.G. (2013). Targeting BTK with ibrutinib in relapsed chronic lymphocytic leukemia. N. Engl. J. Med..

[32] Burger J.A., Tedeschi A., Barr P.M., Robak T., Owen C., Ghia P., Bairey O., Hillmen P., Bartlett N.L., Li J. (2015). Ibrutinib as Initial Therapy for Patients with Chronic Lymphocytic Leukemia. N. Engl. J. Med..

[33] Munir T., Brown J.R., O’Brien S., Barrientos J.C., Barr P.M., Reddy N.M., Coutre S., Tam C.S., Mulligan S.P., Jaeger U. (2019). Final analysis from RESONATE: Up to six years of follow-up on ibrutinib in patients with previously treated chronic lymphocytic leukemia or small lymphocytic lymphoma. Am. J. Hematol..

[34] Honigberg L.A., Smith A.M., Sirisawad M., Verner E., Loury D., Chang B., Li S., Pan Z., Thamm D.H., Miller R.A. (2010). The Bruton tyrosine kinase inhibitor PCI-32765 blocks B-cell activation and is efficacious in models of autoimmune disease and B-cell malignancy. Proc. Natl. Acad. Sci. USA.

[35] Mulder T.A., Peña-Pérez L., Berglöf A., Meinke S., Estupiñán H.Y., Heimersson K., Zain R., Månsson R., Smith C.I.E., Palma M. (2021). Ibrutinib Has Time-dependent On- and Off-target Effects on Plasma Biomarkers and Immune Cells in Chronic Lymphocytic Leukemia. HemaSphere.

[36] Lipsky A.H., Farooqui M.Z., Tian X., Martyr S., Cullinane A.M., Nghiem K., Sun C., Valdez J., Niemann C.U., Herman S.E. (2015). Incidence and risk factors of bleeding-related adverse events in patients with chronic lymphocytic leukemia treated with ibrutinib. Haematologica.

[37] Barf T., Covey T., Izumi R., van de Kar B., Gulrajani M., van Lith B., van Hoek M., de Zwart E., Mittag D., Demont D. (2017). Acalabrutinib (ACP-196): A Covalent Bruton Tyrosine Kinase Inhibitor with a Differentiated Selectivity and In Vivo Potency Profile. J. Pharmacol. Exp. Ther..

[38] Flinsenberg T.W.H., Tromedjo C.C., Hu N., Liu Y., Guo Y., Thia K.Y.T., Noori T., Song X., Aw Yeang H.X., Tantalo D.G. (2020). Differential effects of BTK inhibitors ibrutinib and zanubrutinib on NK-cell effector function in patients with mantle cell lymphoma. Haematologica.

[39] Montoya S., Thompson M.C. (2023). Non-Covalent Bruton’s Tyrosine Kinase Inhibitors in the Treatment of Chronic Lymphocytic Leukemia. Cancers.

[40] Montoya S., Bourcier J., Noviski M., Lu H., Thompson M.C., Chirino A., Jahn J., Sondhi A.K., Gajewski S., Tan Y.S. (2024). Kinase-impaired BTK mutations are susceptible to clinical-stage BTK and IKZF1/3 degrader NX-2127. Science.

[41] Gui F., Jiang J., He Z., Li L., Li Y., Deng Z., Lu Y., Wu X., Chen G., Su J. (2019). A non-covalent inhibitor XMU-MP-3 overrides ibrutinib-resistant BtkC481S mutation in B-cell malignancies. Br. J. Pharmacol..

[42] Woyach J.A., Furman R.R., Liu T.M., Ozer H.G., Zapatka M., Ruppert A.S., Xue L., Li D.H., Steggerda S.M., Versele M. (2014). Resistance mechanisms for the Bruton’s tyrosine kinase inhibitor ibrutinib. N. Engl. J. Med..

[43] Zain R., Vihinen M. (2021). Structure-Function Relationships of Covalent and Non-Covalent BTK Inhibitors. Front. Immunol..

[44] Wang E., Mi X., Thompson M.C., Montoya S., Notti R.Q., Afaghani J., Durham B.H., Penson A., Witkowski M.T., Lu S.X. (2022). Mechanisms of Resistance to Noncovalent Bruton’s Tyrosine Kinase Inhibitors. N. Engl. J. Med..

[45] Wiśniewski K., Puła B. (2024). A Review of Resistance Mechanisms to Bruton’s Kinase Inhibitors in Chronic Lymphocytic Leukemia. Int. J. Mol. Sci..

[46] Bender A.T., Gardberg A., Pereira A., Johnson T., Wu Y., Grenningloh R., Head J., Morandi F., Haselmayer P., Liu-Bujalski L. (2017). Ability of Bruton’s Tyrosine Kinase Inhibitors to Sequester Y551 and Prevent Phosphorylation Determines Potency for Inhibition of Fc Receptor but not B-Cell Receptor Signaling. Mol. Pharmacol..

[47] Blombery P., Thompson E.R., Lew T.E., Tiong I.S., Bennett R., Cheah C.Y., Lewis K.L., Handunnetti S.M., Tang C.P.S., Roberts A. (2022). Enrichment of BTK Leu528Trp mutations in patients with CLL on zanubrutinib: Potential for pirtobrutinib cross-resistance. Blood Adv..

[48] Brown J.R., Desikan S.P., Nguyen B., Won H., Tantawy S.I., McNeely S., Marella N., Ebata K., Woyach J.A., Patel K. (2023). Genomic Evolution and Resistance during Pirtobrutinib Therapy in Covalent BTK-Inhibitor (cBTKi) Pre-Treated Chronic Lymphocytic Leukemia Patients: Updated Analysis from the BRUIN Study. Blood.

[49] Handunnetti S.M., Tang C.P.S., Nguyen T., Zhou X., Thompson E., Sun H., Xing H., Zhang B., Guo Y., Sutton L.A. (2019). BTK Leu528Trp—A Potential Secondary Resistance Mechanism Specific for Patients with Chronic Lymphocytic Leukemia Treated with the Next Generation BTK Inhibitor Zanubrutinib. Blood.

[50] Xu B., Liang L., Jiang Y., Zhao Z. (2024). Investigating the ibrutinib resistance mechanism of L528W mutation on Bruton’s tyrosine kinase via molecular dynamics simulations. J. Mol. Graph. Model..

[51] Veeraraghavan S., Viswanadha S., Thappali S., Govindarajulu B., Vakkalanka S., Rangasamy M. (2015). Simultaneous quantification of lenalidomide, ibrutinib and its active metabolite PCI-45227 in rat plasma by LC–MS/MS: Application to a pharmacokinetic study. J. Pharm. Biomed. Anal..

[52] Gomez E.B., Ebata K., Randeria H.S., Rosendahl M.S., Cedervall E.P., Morales T.H., Hanson L.M., Brown N.E., Gong X., Stephens J. (2023). Preclinical characterization of pirtobrutinib, a highly selective, noncovalent (reversible) BTK inhibitor. Blood.

[53] Nalawansha D.A., Crews C.M. (2020). PROTACs: An Emerging Therapeutic Modality in Precision Medicine. Cell Chem. Biol..

[54] Paiva S.-L., Crews C.M. (2019). Targeted protein degradation: Elements of PROTAC design. Curr. Opin. Chem. Biol..

[55] Pettersson M., Crews C.M. (2019). PROteolysis TArgeting Chimeras (PROTACs)—Past, present and future. Drug Discov. Today Technol..

[56] Rutherford K.A., McManus K.J. (2024). PROTACs: Current and Future Potential as a Precision Medicine Strategy to Combat Cancer. Mol. Cancer Ther..

[57] Hyak J.M., Huang Y., Rogers K.A., Bhat S.A., Grever M.R., Byrd J.C., Kittai A.S., Jones D., Miller C.R., Woyach J.A. (2022). Combined BCL2 and BTK inhibition in CLL demonstrates efficacy after monotherapy with both classes. Blood Adv..

[58] Wierda W.G., Allan J.N., Siddiqi T., Kipps T.J., Opat S., Tedeschi A., Badoux X.C., Kuss B.J., Jackson S., Moreno C. (2021). Ibrutinib Plus Venetoclax for First-Line Treatment of Chronic Lymphocytic Leukemia: Primary Analysis Results from the Minimal Residual Disease Cohort of the Randomized Phase II CAPTIVATE Study. J. Clin. Oncol..

[59] Roy Chowdhury S., Singh A., Nguyen E., Marshall A., Johnston J.B., Gibson S.B., Banerji V. (2019). Ibrutinib in Combination with Venetoclax Decreases Mitochondrial Bioenergetics through the Impaired BTK, AKT and AMPK/SIRT/PGC-1α Signaling Pathway in CLL. Blood.

[60] Tam C.S., Allan J.N., Siddiqi T., Kipps T.J., Jacobs R., Opat S., Barr P.M., Tedeschi A., Trentin L., Bannerji R. (2022). Fixed-duration ibrutinib plus venetoclax for first-line treatment of CLL: Primary analysis of the CAPTIVATE FD cohort. Blood.

[61] Jain N., Keating M.J., Thompson P.A., Ferrajoli A., Burger J.A., Borthakur G., Takahashi K., Estrov Z.E., Sasaki K., Fowler N.H. (2020). Combined Ibrutinib and Venetoclax for First-Line Treatment for Patients with Chronic Lymphocytic Leukemia (CLL): Focus on MRD Results. Blood.

[62] Hillmen P., Rawstron A.C., Brock K., Muñoz-Vicente S., Yates F.J., Bishop R., Boucher R., MacDonald D., Fegan C., McCaig A. (2019). Ibrutinib Plus Venetoclax in Relapsed/Refractory Chronic Lymphocytic Leukemia: The CLARITY Study. J. Clin. Oncol..

[63] Rogers K.A., Woyach J.A. (2024). The evolving frontline management of CLL: Are triplets better than doublets? How will we find out?. Hematol. Am. Soc. Hematol. Educ. Program..

[64] Rogers K.A., Huang Y., Ruppert A.S., Abruzzo L.V., Andersen B.L., Awan F.T., Bhat S.A., Dean A., Lucas M., Banks C. (2020). Phase II Study of Combination Obinutuzumab, Ibrutinib, and Venetoclax in Treatment-Naïve and Relapsed or Refractory Chronic Lymphocytic Leukemia. J. Clin. Oncol..

[65] Woyach J.A., Yin J., Brown J.R., Dinner S., Lozanski G., Little R.F., Miller C., Damarla V.K., Coutre S.E., Ding W. (2023). Results of a phase 3 study of IVO vs IO for previously untreated older patients (pts) with chronic lymphocytic leukemia (CLL) and impact of COVID-19 (Alliance). J. Clin. Oncol..

[66] Davids M.S., Lampson B.L., Tyekucheva S., Wang Z., Lowney J.C., Pazienza S., Montegaard J., Patterson V., Weinstock M., Crombie J.L. (2021). Acalabrutinib, venetoclax, and obinutuzumab as frontline treatment for chronic lymphocytic leukaemia: A single-arm, open-label, phase 2 study. Lancet Oncol..

[67] Jain N., Ferrajoli A., Swaminathan M., Reville P.K., Burger J.A., Bharathi V., Atluri H., Cherng H.-J.J., Bataller A., Jabbour E. (2024). Combined Pirtobrutinib, Venetoclax, and Obinutuzumab As First-Line Treatment of Patients with Chronic Lymphocytic Leukemia (CLL). Blood.

[68] Buhimschi A.D., Armstrong H.A., Toure M., Jaime-Figueroa S., Chen T.L., Lehman A.M., Woyach J.A., Johnson A.J., Byrd J.C., Crews C.M. (2018). Targeting the C481S Ibrutinib-Resistance Mutation in Bruton’s Tyrosine Kinase Using PROTAC-Mediated Degradation. Biochemistry.

[69] Salvaris R.T., Brennan J., Lewis K.L. (2025). BTK Is the Target That Keeps on Giving: A Review of BTK-Degrader Drug Development, Clinical Data, and Future Directions in CLL. Cancers.

[70] Huynh T., Rodriguez-Rodriguez S., Roleder C., Whelan S., Tan M., Lee E., Munson P., Danilov A.V. (2024). Nx-2127 and Nx-5948, Two Clinical Stage Cereblon-Recruiting BTK Degraders, Facilitate T Cell Functionality in Chronic Lymphocytic Leukemia. Blood.

[71] Robbins D.W., Kelly A., Tan M., McIntosh J., Wu J., Konst Z., Kato D., Peng G., Mihalic J., Weiss D. (2020). Nx-2127, a Degrader of BTK and IMiD Neosubstrates, for the Treatment of B-Cell Malignancies. Blood.

[72] Fiskus W.C., Das K., Mill C.P., Birdwell C.E., Davis J.A., Alhamadani N., Philip K., Tan M., Brown R.A., Green M.R. (2022). Bruton’s Tyrosine Kinase (BTK) Degrader Nx-2127 Exhibits Lethal Activity and Synergy with Venetoclax and BET Protein Inhibitor Against MCL Cells Sensitive or Resistant to Covalent BTK Inhibitors. Blood.

[73] Wong R.L., Choi M.Y., Wang H.-Y., Kipps T.J. (2024). Mutation in Bruton Tyrosine Kinase (BTK) A428D confers resistance To BTK-degrader therapy in chronic lymphocytic leukemia. Leukemia.

[74] Nawaratne V., Sondhi A.K., Abdel-Wahab O., Taylor J. (2024). New Means and Challenges in the Targeting of BTK. Clin. Cancer Res..

[75] Bonfiglio S., Sutton L.-A., Ljungström V., Capasso A., Pandzic T., Weström S., Foroughi-Asl H., Skaftason A., Gellerbring A., Lyander A. (2023). BTK and PLCG2 remain unmutated in one-third of patients with CLL relapsing on ibrutinib. Blood Adv..

[76] Brown J.R., Li J., Eichhorst B.F., Lamanna N., O’Brien S.M., Tam C.S., Qiu L., Ramakrishnan V., Huang R., Shi Y. (2023). Acquired Mutations in Patients (Pts) with Relapsed/Refractory (R/R) Chronic Lymphocytic Leukemia (CLL) That Progressed in the ALPINE Study. Blood.

[77] Woyach J., Huang Y., Rogers K., Bhat S.A., Grever M.R., Lozanski A., Doong T.-J., Blachly J.S., Lozanski G., Jones D. (2019). Resistance to Acalabrutinib in CLL Is Mediated Primarily By BTK Mutations. Blood.

[78] Palma M., Mulder T.A., Österborg A. (2021). BTK Inhibitors in Chronic Lymphocytic Leukemia: Biological Activity and Immune Effects. Front. Immunol..

[79] Lévy V., Delmer A., Cymbalista F. (2021). Frontline treatment in CLL: The case for time-limited treatment. Hematology.

[80] Rhodes J.M., Lopez C.A., Barrientos J.C. (2023). MRD-directed therapy in CLL: Ready for prime time?. Hematology.

[81] Benintende G., Pozzo F., Innocenti I., Autore F., Fresa A., D’Arena G., Gattei V., Lurenti L. (2023). Measurable residual disease in chronic lymphocytic leukemia. Front. Oncol..

[82] Rogers K.A., Woyach J.A. (2022). A CAPTIVATE-ing new regimen for CLL. Blood.

[83] Böttcher S., Ritgen M., Pott C., Brüggemann M., Raff T., Stilgenbauer S., Döhner H., Dreger P., Kneba M. (2004). Comparative analysis of minimal residual disease detection using four-color flow cytometry, consensus IgH-PCR, and quantitative IgH PCR in CLL after allogeneic and autologous stem cell transplantation. Leukemia.

[84] Raponi S., Della Starza I., De Propris M.S., Del Giudice I., Mauro F.R., Marinelli M., Di Maio V., Piciocchi A., Foà R., Guarini A. (2014). Minimal residual disease monitoring in chronic lymphocytic leukaemia patients. A comparative analysis of flow cytometry and ASO IgH RQ-PCR. Br. J. Haematol..

[85] Dogliotti I., Drandi D., Genuardi E., Ferrero S. (2018). New Molecular Technologies for Minimal Residual Disease Evaluation in B-Cell Lymphoid Malignancies. J. Clin. Med..

[86] Rawstron A.C., Kreuzer K.-A., Soosapilla A., Spacek M., Stehlikova O., Gambell P., McIver-Brown N., Villamor N., Psarra K., Arroz M. (2018). Reproducible diagnosis of chronic lymphocytic leukemia by flow cytometry: An European Research Initiative on CLL (ERIC) & European Society for Clinical Cell Analysis (ESCCA) Harmonisation project. Cytom. Part B Clin. Cytom..

[87] Winter A.M., Landever O., Mendries H., Van Heeckeren W., Fu C.-L., Dean R.M., Brooks T.R., Jagadeesh D., Caimi P.F., Hill B.T. (2024). Real World Experience with Time Limited Venetoclax and Obinutuzumab (VO) for Frontline Treatment of CLL/SLL with MRD Determination By Clonoseq^®^. Blood.

[88] Chandhok N.S., Sekeres M.A. (2025). Measurable residual disease in hematologic malignancies: A biomarker in search of a standard. eClinicalMedicine.

